# Trio-Based Deep Sequencing Reveals a Low Incidence of Off-Target Mutations in the Offspring of Genetically Edited Goats

**DOI:** 10.3389/fgene.2018.00449

**Published:** 2018-10-04

**Authors:** Chao Li, Shiwei Zhou, Yan Li, Guanwei Li, Yige Ding, Lan Li, Jing Liu, Lei Qu, Tad Sonstegard, Xingxu Huang, Yu Jiang, Yulin Chen, Bjoern Petersen, Xiaolong Wang

**Affiliations:** ^1^College of Animal Science and Technology, Northwest A&F University, Yangling, China; ^2^Life Science Research Center, Yulin University, Yulin, China; ^3^Recombinetics, Saint Paul, MN, United States; ^4^School of Life Sciences and Technology, ShanghaiTech University, Shanghai, China; ^5^Institut für Nutztiergenetik, Friedrich-Loeffler-Institut, Neustadt an der Weinstraße, Germany

**Keywords:** genome editing, CRISPR/Cas9, whole genome sequencing, off-target, *de novo* mutation

## Abstract

Unintended off-target mutations induced by CRISPR/Cas9 nucleases may result in unwanted consequences, which will impede the efficient applicability of this technology for genetic improvement. We have recently edited the goat genome through CRISPR/Cas9 by targeting *MSTN* and *FGF5*, which increased muscle fiber diameter and hair fiber length, respectively. Using family trio-based sequencing that allow better discrimination of variant origins, we herein generated offspring from edited goats, and sequenced the members of four family trios (gene-edited goats and their offspring) to an average of ∼36.8× coverage. This data was to systematically examined for mutation profiles using a stringent pipeline that comprehensively analyzed the sequence data for *de novo* single nucleotide variants, indels, and structural variants from the genome. Our results revealed that the incidence of *de novo* mutations in the offspring was equivalent to normal populations. We further conducted RNA sequencing using muscle and skin tissues from the offspring and control animals, the differentially expressed genes (DEGs) were related to muscle fiber development in muscles, skin development, and immune responses in skin tissues. Furthermore, in contrast to recently reports of Cas9 triggered p53 expression alterations in cultured cells, we provide primary evidence to show that Cas9-mediated genetic modification does not induce apparent p53 expression changes in animal tissues. This work provides adequate molecular evidence to support the reliability of conducting Cas9-mediated genome editing in large animal models for biomedicine and agriculture.

## Introduction

Recent advances in genome editing using the type II bacterial clustered, regularly interspaced, palindromic repeats (CRISPR)-associated (Cas) system have enabled efficient genetic modification in the genomes of many organisms, including large animal models for biomedicine or agricultural purposes. Within the CRISPR/Cas9 system, the Cas9 from *Streptococcus pyogenes* recognizes a 5′-NGG-3′ PAM sequence on the non-target DNA strand, and allows complementation for 20-base-pair of target DNA sequence (Mali and Church, 2013). However, unwanted off-target mutations and chromosomal translocations are potential drawbacks, raising concerns about the precision of the CRISPR/Cas system, which would prohibit its use in correcting human genetic diseases, and for optimal commercialization within livestock genetic improvement programs (Garas et al., 2015; Ruan et al., 2017).

Off-target detection in advance is a challenge, as the existing proven analysis methods depend largely on amplification and sequencing of pre-selected off-target sites, identified by several bioinformatics tools [e.g., CasOT (Xiao et al., 2014), and CT-Finder (Zhu et al., 2016)]. This approach can be more difficult to implement when the analysis aims to interrogate all possible non-unique matches and allowed mismatches distal from the PAM sequences. Compared with Sanger sequencing or short-reads deep sequencing of pre-selected off-target PCR amplicons, whole genome sequencing (WGS) is a less biased assessment of off-target mutations caused by Cas9. WGS is able to fully characterize the genome-wide mutation profiles, which not only include small insertion and deletions (indels) and SNPs but also structural variants such as inversions, rearrangements, duplications, and major deletions (Zischewski et al., 2017). This approach has been used to screen for off-target mutations induced by CRISPR/Cas9 in human cells (Smith et al., 2014; Kim et al., 2015), mice (Veres et al., 2014; Iyer et al., 2015), and plants (Zhang et al., 2017). Screening for off-target mutagenesis in gene-edited animals is rare and will be highly important in farm animal since gene editing brings the commercial benefits of improving the genetics of livestock, and also serves as a research model for biomedical studies. In addition, investigating the mutation profiles in the offspring of edited animals will provide fundamental evidence to support the reliability of the CRISPR/Cas9 system.

Genetically modified goats were successfully generated through multiplex injection of four sgRNAs targeting two functional genes (*MSTN* and *FGF5*) and Cas9 mRNA in one-cell-stage embryos (Wang et al., 2015). The desired phenotypes were independently observed in the edited animals. For example, disruption of *MSTN* caused increased muscle mass (Wang et al., 2018b), while disruption of *FGF5* increased the number of secondary hair follicles and enhanced fiber length (Wang et al., 2016). Although healthy edited goats with ideal phenotypes were generated by this effort, the rate of genome-wide off-target mutations in the edited animals and their progenies have not been well documented. As such, 11 goats from four family trios were sequenced at a high coverage (>36.8×), and the mutational profiles of these animals were systematically characterized to determine rates of off-target activity. The *de novo* mutation rate in the offspring were determined to be largely equivalent to the mutation rates of other populations such as human and cattle. Together with our previous results using trio-based WGS to show a low incidence of off-target mutations in gene-edited sheep (Wang et al., 2018a), this work confirms the reliability of a multiplex-based CRISPR/Cas9 approach for the production of offspring from genetically modified large animal models that are intended for bio-medical studies and food production.

## Materials and Methods

### Animals

**FIGURE 1:**
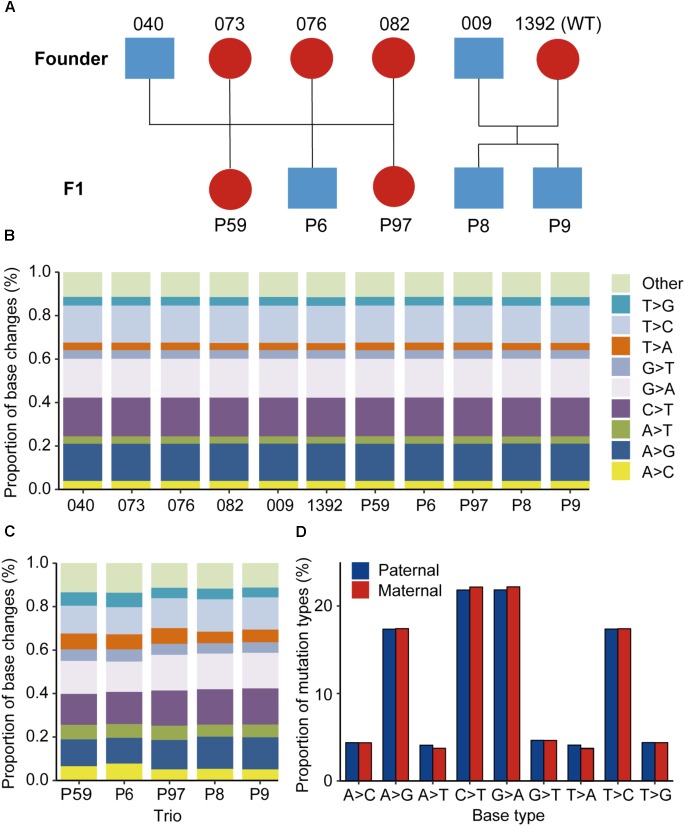
Animals used and the mutational spectra in the edited goat genome. **(A)** Animals used in this study. **(B)** Proportion of base changes in each animal in this study. Blue color represents male animals, while red color represents female animals. **(C)** Proportion of base changes in each family trio. **(D)** Nucleotide substitution ratios from paternal or maternal origins in the F1 progenies.

The genetically edited animals (Shaanbei Cashmere goat) used in the present study were generated previously (Wang et al., 2015). Two edited males (#009 and #040) were selected and mated with either edited (#076, #082, #073) or wildtype (#1392) female animals (same breed) after puberty (1.5 years old). All animals involved in this study were maintained at the Research Farm of Yulin University, Yulin, China. All protocols involving animals were approved by the College of Animal Science and Technology, Northwest A&F University (Approval ID: 2014ZX08008002). The pedigree information of these four family trios was validated by estimating the kinship coefficient according to a previous study (Manichaikul et al., 2010), and were present in **Figure 1A**. Additional WGS data from two goat trios were selected as control trios for the present study (unpublished data).

### Whole-Genome Sequencing and Data Analyses

A total of 11 goats from four family trios were chosen for WGS (**Figure 1A**). For each animal, genomic DNA was extracted from peripheral venous blood samples with a Qiagen DNeasy Blood and Tissue Kit (Qiagen). To construct the WGS library, 1 μg of genomic DNA was fragmented to around 300 bp by ultrasonication using a Covaris S2 system. Then, the sheared DNA fragments were used for library construction using an Illumina TruSeq DNA library preparation kit at Novogene^1^. The final quality-ensured libraries were sequenced on an Illumina Hiseq 3000 for 125-bp paired-end reads. The raw sequencing reads were first filtered to remove low quality paired reads with the following criteria: (1) reads with >10% N bases, (2) reads with >50% bases with a sequencing quality of <3, and (3) reads with residual length of <40 bases after the adaptor sequences were trimmed. All reads that passed the quality control procedures were converted into FASTQ files.

To analyze the mutational classes in all the mutations, 12 different mutations were categorized into nine classes (T > G, T > C, T > A, G > T, G > A, C > T, A > T, A > G, and A > C), and the base changes were measured in each animal and each family trio (Jónsson et al., 2017).

### Identification of *de novo* Variants

All reads after quality control were mapped to the goat reference sequence assembly ARS1 (Bickhart et al., 2017), using BWA (v0.7.13) tools (Li and Durbin, 2009) with default parameters. Local realignment and base quality re-calibration were conducted using the Genome Analysis Toolkit (GATK, v.2.7-2) (Mckenna et al., 2010). Both single nucleotide variants (SNVs) and indels (2–100 bp) were called using GATK and Samtools (Li et al., 2009; Mckenna et al., 2010).

*De novo* SNVs and indels for each F1 animal were extracted according to the following criteria: (1) SNVs were independently identified by GATK and Samtools within a single trio, (2) choosing the overlapped SNVs that were identified by GATK and Samtools, and selecting the SNVs that were specific in each F1 animal; (3) filtering SNVs that exist in a goat SNP database (*n* = 234, 11 populations including 30 cashmere goats, >79 million SNPs, unpublished data); (4) filtering SNVs with a read depth in parents <12, and in F1 animals <1/10 of the sum of the coverage in both parents (Allen et al., 2013; Jónsson et al., 2017); (5) the normalized phred-scaled likelihood (PL) scores for the genotypes (AA, AB, and BB) of F1 animals (A, reference allele; B, alternate allele), the PL scores for each genotype in founders should be >20, 0, and >0, the PL scores for each genotype in both parents should be 0, >20, and >20 (Allen et al., 2013); (6) filtering SNVs with >10% average soft clipping per read; (7) manual examination to remove mis-aligned or miscalled SNVs/indels. Copy number variations (CNVs) were identified using a CNVcaller tool (Wang X. et al., 2017), we developed recently. The scanning window was defined as 400 bp, and the effective window value was set as 7, then the low-quality CNVs were removed manually. Structural variations (SVs) were independently detected using BreakDancer (Chen et al., 2009) and Lumpy (Layer et al., 2014) with suggested parameters. The detailed filtering pipelines for *de novo* SNVs are summarized in **Table 1**.

**Table 1:** Summary of filtering process for SNVs and indels in the F1 progenies.

Variants	P59	P6	P97	P8	P9
***SNVs***					
Called by GATK	11,809,830	11,863,185	11,764,624	11,649,340	11,665,474
Called by Samtools	12,188,260	12,213,943	12,130,082	12,021,749	12,031,956
GATK+Samtools	1404	1461	9610	5839	7099
Filtering using the goat SNP databases (*n* = 233, >79 million SNPs)	1081	1174	3018	1526	1818
Reads depth and allelic balance filtering	136	166	166	139	127
After PL scores, soft clipping, and ambiguous bases filtering	53	70	68	50	42
After manual check	18	18	24	11	14
Validated by Sanger sequencing	11/15	7/9	–	–	5/7
Mutation rate (×10^-8^)	1.08	1.42	1.38	1.02	0.85
***Indels***				
Calledby GATK	1,796,995	1,813,872	1,781,374	1,706,893	1,713,903
Called by Samtools	1,579,898	1,589,589	1,546,312	1,408,611	1,420,070
GATK+Samtools	815	898	3314	1198	1583
Filtering using the goat SNP databases (*n* = 233, >79 million SNPs)	187	233	562	181	222
Reads depth and allelic balance filtering	20	29	26	16	22
After PL scores, soft clipping, and ambiguous bases filtering	7	14	7	2	7
After manual check	3	8	5	1	2
Validated by Sanger sequencing	1/2	5/8	4/5	–	2/2

### Prediction of Off-Target Sites

The putative off-target sites in the goat genome that might be recognized by the sgRNAs targeting the *MSTN* and *FGF5* genes were predicted by CasOT (Xiao et al., 2014), and Cas-OFFinder (Bae et al., 2014). The potential off-target sites were defined as up to five mismatches according to a recent study (Boyle et al., 2017). The detailed information of predicted off-target sites is summarized in **Supplementary Table S1**.

### Estimation of Mutation Rate

The estimation of mutation rate per base pair per generation was calculated according to a recent study (Jónsson et al., 2017). Briefly, we retrieved short read sequences (.bam file) were retrieved by averaging the coverage in 10,000 base windows and the sequences from autosome genome within 13× to 130× coverage were selected. This resulted in 245,722 effective windows or 2,457,220,000 base pairs (R) within our coverage range. We then estimated the mutation rate per base pair per generation for each F1 animal by dividing the average number of *de novo* mutations (μ_α_) by twice the R account.

$${}_{\mu }^{\wedge }g=\frac{{}_{\mu }^{\wedge }\alpha }{2\times R}$$

### Validation of Edited Sites, SNVs, and Indels

PCR-based Sanger sequencing was conducted to validate the genetic regions with editing or the existence of *de novo* variants (SNVs or indels) identified by WGS. Primers for amplifying the edited sites or the regions encompassing *de novo* variants are listed in **Supplementary Tables S2, S3**. Procedures for the purification of PCR products, the T7E1 cleavage assay, and Sanger sequencing were conducted according to our previously report (Wang et al., 2015).

### RNA Sequencing and Data Analysis

Muscle from *MSTN*-edited and skin tissues from *FGF5*-edited F1 animals (*n* = 3) and wild-type animals (*n* = 3) at the same age (4-month) on the same farm, were collected for RNA-seq analysis. RNA extraction and sequencing were performed as described previously (Wang L. et al., 2017). Total RNA was isolated using Trizol Reagent (Invitrogen) and then treated with RNAse-free DNase I (Qiagen) according to the manufacturer’s instructions. The quality and concentration of the total RNA were determined using an Agilent 2100 Bioanalyzer (Agilent). From each sample, 12 RNA libraries were constructed and oligo (dTs) were used to isolate poly (A) mRNA. The mRNA was fragmented and reverse transcribed using random primers. Second-strand cDNAs were synthesized using RNase H and DNA polymerase I. The double-strand cDNAs were then purified using the QiaQuick PCR extraction kit. The required fragments were purified via agarose gel electrophoresis and were enriched through PCR amplification. Finally, the amplified fragments were sequenced using Illumina HiSeq^TM^ 3000 system at Novogene (see text footnote 1), according to the manufacturer’s instructions.

Among the raw data from RNA-seq, the sequencing adaptors, reads with unknown nucleotides larger than 5%, and the bases with low quality (more than half of the bases’ qualities were less than 10) were removed. The remaining clean data was mapped to the currently available goat genome sequence assembly (ARS1) (Bickhart et al., 2017) using TopHat2 (Kim et al., 2013), to screen differentially expressed genes (DEGs). Counts for each gene were computed by means of the HTSeq Python package (Anders et al., 2015), and DEGs between the F1 progenies and control groups were determined with the EdgeR Bioconductor package using the classic method (Robinson et al., 2010). Gene Ontology (GO) functional enrichment analysis was conducted using g: Profiler to identify the functional categories enriched in DEGs (Reimand et al., 2016). The default settings were used, and GO terms with corrected *P*-value of less than 0.05 were considered significantly enriched.

The same RNA from muscle or skin tissues was used for qPCR analyses, to validate the RNA-seq results. First strand cDNA synthesized using the Thermo Scientific RevertAid First Strand cDNA Synthesis kit (#K1622, Thermo Fisher Scientific, United States) under the manual instructions, and was then subjected to quantification using a standard SYBR Premix Ex Taq (Tli RNaseH Plus) kit (#RR420A, Takara, China) on the Bio-Rad CFX96 Real-Time System. The primers for eight genes used for this study are listed in **Supplementary Table S4**. Biological and technical replicates were performed in triplicate for each sample. Gene relative expression was calculated using 2^-ΔΔCt^ method, quantified relative to the housekeeping gene *GAPDH*.

### Immunofluorescent Staining and Western Blotting

The biopsied tissues were immediately fixed in 4% paraformaldehyde at 4°C overnight. The fixed tissues were then embedded in paraffin using standard immunohistochemical protocols. The immunofluorescence staining was conducted with anti-p53 (Cell Signaling Technology) primary antibody, the sections were then counterstained with Hoechst 33342 and analyzed by confocal laser microscopy. We extracted total protein from muscles, and then quantified the protein using the Bradford assay. Equal amounts of soluble protein were separated by SDS/PAGE and transferred onto a polyvinylidene difluoride membrane (PVDF, Roche). Immunoblotting was conducted using antibodies specific for phospho-p53 (Ser15) (Cell Signaling Technology, 1:1000), and β-actin (Proteintech, 1:1000). Primary antibodies were visualized using a fluorescence imager system (Sagecreation).

## Results

### Generation of F1 Progenies

Cas9 mRNA and single guide RNAs targeting two functional genes, *MSTN* and *FGF5*, were multiplex-injected into one-cell-stage goat embryos, to generate animals with gene disruption (Wang et al., 2015). From this treatment, edited goats with improved phenotypes for muscling and fiber length were successfully obtained (Wang et al., 2016, 2018b). We then selected five founder animals (#9, #040, #076, #082, and #073) and one wildtype individual (#1392) for natural breeding, and obtained five F1 progenies (**Figure 1A**). We next genotyped the targeted sites in the progenies and their parents through Sanger sequencing. The genotypes of mutations at on-targeting site were validated by both WGS data (**Supplementary Figure S1**) and Sanger sequencing (**Supplementary Figure S2**). The sequencing data confirmed that the edited sites in the founder animals are successfully transmitted to their offspring, except the F1 animal #P59 was wild-type at the *MSTN*_sg1 locus, most likely because its dam is wildtype at this site (**Supplementary Figure S2**). In particular, mutations in #009 were transmitted to the twin progeny #P8 and #P9, even though #009 was mated with a wildtype female goat.

### Deep Sequencing of Family-Trio Individuals

These 11 animals representing four family trios were subject to WGS variant analyses. The kinship coefficient values in each animal was used to ensure the correct pedigree information (**Supplementary Table S5**). WGS of 11 animal genomic DNAs yielded a total of 722 Gb of raw data, and produced between 516 and 944 million sequence reads mapped per animal (**Supplementary Table S6**). Over 99.02% of the generated sequence reads were mapped, indicating that high quality sequences were obtained. After alignment to the goat reference genome (ARS1) (Bickhart et al., 2017), an average of ∼36.8× (25.0–47.8×) sequencing depth were generated for further analysis (**Supplementary Table S6**).

Of all the SNVs identified by GATK, the mutation spectrums in each animal and each trio were analyzed. It was found that the C > T, A > G, G > A, and T > C substitutions are predominant in all the mutation types in each sequenced animal, and each base change type represents >17% of base changes (**Figure 1B**). Additionally, the proportion of base changes in parents and offspring is non-significant in each trio (*p* = 0.326, Student’s *t*-test) (**Supplementary Figure S3**). The base changes in each family trio were further examined, and no significant changes were found among the trios used for sequencing (**Figure 1C**). Moreover, we observed that the differences in nucleotide substitution patterns between paternal and maternal mutations in F1 animals were non-significant (**Figure 1D**).

### Identification and Validation of *de novo* SNVs

**FIGURE 2:**
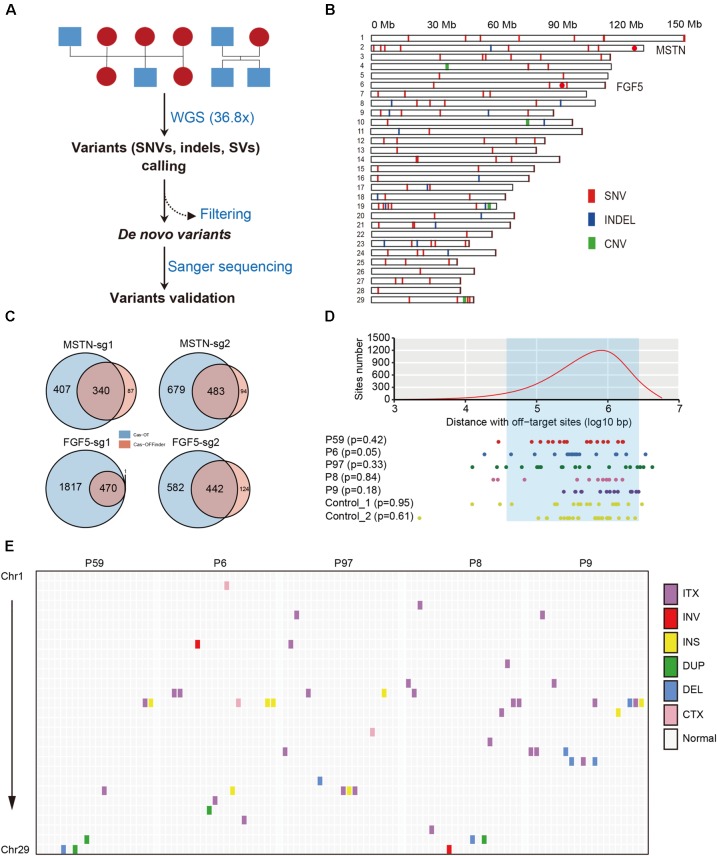
Structural features of *de novo* mutations in the goat genome. **(A)** Workflow of filtering procedures of *de novo* variants in goat family trios. **(B)** Genomic distribution of *de novo* variants (SNVs, indels, and CNVs) in the goat genome. Red dots indicate the location of the two target sites in *MSTN* and *FGF5*. **(C)** Summary of off-target sites predicted by CasOT and Cas-OFFinder at each target site. **(D)** The distances between 100,000 randomly selected sites (upper), and *de novo* SNVs (below) to predicted off-target sites. The off-target sites were defined as one mismatch at seed regions, and up to four mismatches at non-seed regions. The least distance to predicted off-target sites was chosen. The area between two dashlines represents the 95% confidence interval. **(E)** Genomic distribution of SVs across the goat genome.

To detect SNVs that may be derived from Cas9-mediated genetic modification, we employed a series of stringent variant filtering procedures (**Figure 2A** and **Table 1**). We initially called >11.6 million SNVs by GATK, and >12.0 million SNVs by Samtools independently in each progeny. We then selected the specific SNVs in each F1 animal, and chose the SNVs that were both identities by GATK and Samtools. Next, any SNVs that already existed in the goat SNP database (294 individuals from 11 wild and domestic populations, >79 million SNPs) were removed, and further filtering procedures included read depth, phred-scaled likelihood (PL) scores and manual examination according to a previous study (Allen et al., 2013). After manual check, 18, 24, 18, 11, and 14 SNVs remained in the F1 progenies P6, P97, P59, P8, and P9, respectively (**Table 1** and **Supplementary Table S4**). These *de novo* SNVs were distributed in all chromosomes, and did not no cluster near the gene target sites (**Figure 2B**). We selected the SNVs from P6, P59, and P9 for PCR amplification followed by Sanger sequence validation, which confirmed that over 70% of SNVs truly exist (**Table 1** and **Supplementary Table S2**). The germline *de novo* mutation rate (per base pair per generation) in these F1 progenies was estimated to range from 0.85 × 10^-8^–1.42 × 10^-8^ base substitutions per site per generation (**Table 1**). We next predicted the genome-wide off-target sites using two programs, Cas-OT and Cas-OFFinder, we show that the vast majority of the off-targets identified by Cas-OFFinder were also included in the off-targets predicted by Cas-OT (**Figure 2C**). To ensure the reliability of predicted off-targets, we chose the overlapped off-targets for further analysis. The distance between 100,000 randomly selected SNV sites, and *de novo* SNVs to 534 predicted off-target sites were simulated (five mismatches) (**Supplementary Table S1** and **Supplementary Figure S4**), and no significant effects between the random selected SNVs and these *de novo* SNVs were observed from five F1 progenies, and two F1 animals from the control trios (*p* > 0.05, Kolmogorov–Smirnov test) (**Figure 2D**). Together, these results indicated that *de novo* SNVs in the F1 progenies resulted from normal spontaneous mutagenesis rather than from CRISPR/Cas-mediated gene editing.

### Identification of Indels, CNVs, and SVs

Next, a comparative analysis within each trio was performed to identify for Cas9-induced small indels, given the possible likely outcomes of Cas9 induced double-strand breaks (DSB) repaired via non-homologous end-joining (NHEJ). Similar filtering procedures were used to screen candidate *de novo* indels as were conducted for *de novo* SNVs. After stringent indel filtering procedures including read depth, PL value and manual examination, a total of 19 indels were determined as *de novo* indels in all the five F1 animals (**Table 2** and **Supplementary Table S3**). PCR-based Sanger sequencing confirmed the existence of 13 indels (**Table 1** and **Supplementary Table S3**).

**Table 2:** Identified candidate CNV and SVs in the F1 progenies.

Variants	P59	P6	P97	P8	P9
***CNV***					
Called by CNVcaller	1426	1016	2835	418	307
Filtering by genotype and effective window	6	8	15	5	2
Candidate *de novo* CNVs	1	1	2	0	0
***SVs***					
(1) Called by BreakDancer, and specific in F1 animals	1570	1411	1633	2058	2107
After removal of common SVs in every two animals, and the read depth <50%, manual check	3	9	13	12	13
(2) Called by Lumpy	3609	3116	3406	2697	3082
After removal of common SVs in every two animals, and the read depth <50%, manual check	3	2	0	3	1

We next examined whether the large-scale genomic alterations (CNVs and SVs) could be attributed to Cas9 nucleases. CNVcaller (Wang X. et al., 2017) was used to search for CNVs, and after filtering CNVs by its genotypes and the effective window value, only four candidate CNVs were left in the F1 animals (**Figure 2B** and **Table 2**). A number of SVs were identified using BreakDancer (Chen et al., 2009) and Lumpy (Layer et al., 2014), and only a few remained after filtering and were considered as candidate *de novo* SVs in each animal (**Figure 2E**, **Table 2**, and **Supplementary Table S7**).

### Analyzing of Off-Target Mutations

To assess the off-target effects in F1 animals, we identified, using Cas-OFFinder, potential off-target sites with up to 3-nucleotide mismatches and NRG PAM sites in the goat genome. We then determined whether the *de novo* mutations, as well as the mutations that shared in parents and progenies were within the potential off-target sites, merely two indels were determined as off-target mutations (**Supplementary Table S8**). Sanger sequencing further validated these two variants (**Supplementary Figure S5**), indicating the off-target mutations are low in the offspring of edited animals and is guide RNA specific.

### RNA-seq Analyses of Edited Animals

**FIGURE 3:**
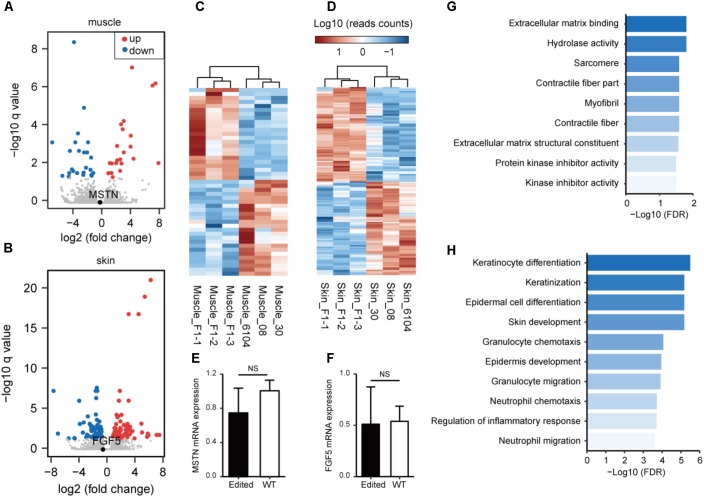
Transcriptome analysis of gene targeting in the edited and WT animals. A volcano plot of gene expression patterns is shown in the edited and WT animals using muscle **(A)** or skin **(B)** tissues. **(C)** Heatmap of DEGs demonstrating unsupervised hierarchical clustering of *MSTN*-edited and WT animals in muscle tissues. **(D)** Heat maps of DEGs demonstrating unsupervised hierarchical clustering of *FGF5*-edited and WT animals in muscle tissues. Quantitative RT-PCR validation of the mRNA expression of *MSTN* in muscle **(E)**, and *FGF5* in skin tissues **(F)**. Enriched Gene Ontology terms using DEGs revealed in muscle **(G)** or skin **(H)** tissues.

We have recently analyzed the transcriptome profiles using muscle tissues from *MSTN* and/or *FGF5*-edited cashmere goats (Wang L. et al., 2017), and showed that the *MSTN*-disruption resulted in substantial changes in genes involved in lipid metabolism and biosynthesis. To better understand the transcriptional consequences of gene disruption in the genome of F1 progenies, we conducted transcriptome sequencing (RNA-seq) analysis in the edited progenies and WT animals using muscle or skin tissues. The volcano plot demonstrated that the expression of *MSTN* did not change significantly (**Figure 3A**). However, the disruption of *MSTN* resulted in 43 (23 up-regulated and 20 down-regulated) genes with significantly changed expression (**Figure 3C** and **Supplementary Table S9**). Some of these genes are known to be associated with muscle developmental including *FMOD*, *ARG2*, *TNMD*, *CSRP3*, *PCK2*, *EGR1*, and *TNC*. Meanwhile, disruption of *FGF5* led to the identification of 140 DEGs (74 up-regulated and 66 down-regulated) in the skins of F1 progenies and control animals (**Figures 3B,D** and **Supplementary Table S10**). Key regulators related to skin/hair follicle development such as *AQP3*, *AQP5*, *SPINK7*, and *WIF1* were involved, indicating that *FGF5* disruption may stimulate hair follicle functions resulting in increased fiber length (Wang et al., 2016). We performed qPCR on ten DEGs (including *MSTN* and *FGF5*) using RNA isolated from tissues of the same individuals. The validation results revealed a similar correlation between the RNA-seq and qPCR results (**Figures 3E,F** and **Supplementary Figure S6**), suggestion the reliability of RNA-seq analyses.

Subsequently, we performed GO enrichment analysis to predict the over-represented GO terms associated the DEGs identified in muscle and/or skin tissues. The DEGs identified in muscles exhibited significant over-representation of GO terms related to muscle fiber development (such as “sarcomere,” “contractile fiber,” and “myofibril”) (**Figure 3G**). The DEGs identified in skins are significantly more enriched in GO terms related to skin and hair follicle development (“skin development,” “keratinization,” “keratinocyte differentiation,” and “epidermis development”) and immune responses (“granulocyte chemotaxis and migration,” “leukocyte chemotaxis and migration,” and “myeloid leukocyte migration”) (**Figure 3H**).

**FIGURE 4:**
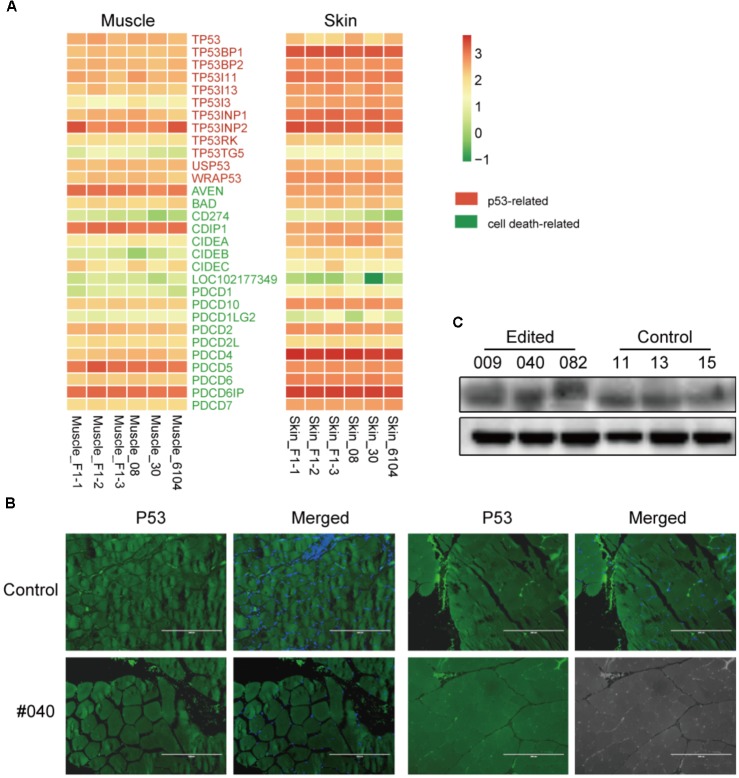
p53 dependent expression in the muscles of edited animals and controls. **(A)** Heatmap of p53 and cell death related genes in F1 progenies and WT animals in muscle and skin tissues. **(B)** Immunofluorescence staining of muscles from a mutant (#040) and a control animal (#11) using anti-p53 antibodies (green) and counterstained with Hoechst 33342 (blue). Scale bar, 200 μm. **(C)** Western blot analysis using anti-p53 and anti-β-actin (control) antibodies in mutant (#009, #040, and #082) and three controls (#11, #13, and #15).

Since two recent studies described that CRIPSR/Cas9-mediated genetic modification in normal cultured cells derived a p53-dependent toxic response (Haapaniemi et al., 2018; Ihry et al., 2018), which may raise tumor risks. To validate this scenario in edited animals, we first investigated the expression of p53 and cell death-related genes retrieved from the RNA-seq data in skin and muscles, no significant gene expression changes were observed (**Figure 4A**). We further assessed the protein expression of p53 in the muscle of *MSTN*-disrupted animals. The immunofluorescence staining indicated no apparent changes of p53 expression in muscles between an edited animal (#040) and controls (#11) (**Figure 4B**). Western blotting further verified the p53 expression in muscles (**Figure 4C**). These results are consistent with the p53 expression in mice (personal communication), and suggested that Cas9-mediated modification did not induce apparent p53 expression changes in animal tissues. This result was probably due to p53-dependent molecular response in animal bodies may be differed with that in cultured cells (Haapaniemi et al., 2018), or the cellular response to DNA damage was repaired during the embryonic development process.

## Discussion

Deep sequencing is able to fully characterize the mutational profiles in genomes, and is used to detect mutational changes in genetically edited organisms (Huang et al., 2016). In this study, through sequencing four family trios at a high coverage, the *de novo* variants in F1 animals that could be attributed to the engineered nucleases were determined to be neglectable, representing a low incidence of CRISPR/Cas9-mediated off-target mutations. On the other hand, our results further demonstrate the reliability of WGS in documenting mutations induced by genome editing.

Previous studies have shown that the *de novo* mutations exhibited variant type preferences and discriminative parental origins, and the mutational signatures were influenced by multiple factors including nucleotide type, sequence context, replication timing, and epigenetics. In this study, the mutational spectrum in genomes other than human and mice are reported. The C-T and A-G transitions were predominant in goat genomes, the enrichment of C > T transitions at CpG dinucleotides could reflect spontaneous domination of methylated cytosine to thymine (Bolli et al., 2014). We observed that the proportion of base changes was not significant in the parents or in the F1 progenies (**Figure 1C**), indicating the mutation profiles are largely stable in the edited animals and their offspring.

**FIGURE 5:**
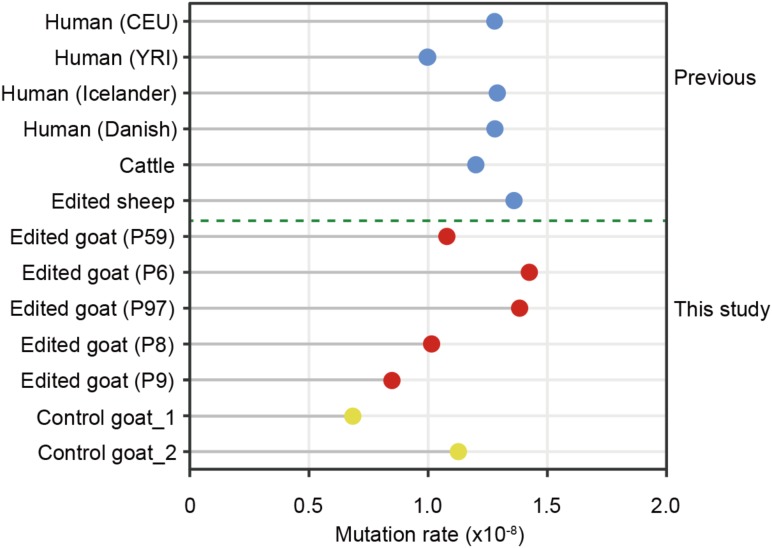
The mutation rates in edited goats and other populations.

Mutation rate is a key parameter for calibrating the timescale of sequence divergence. The estimated average germline *de novo* SNV rate (per base pair per generation) in the present study is 1.15 × 10^-8^, which is equivalent to the average germline mutation rate in serval trio-based human populations including CEU (1.2 × 10^-8^) and YRI (1.0 × 10^-8^) (Consortium et al., 2010), Icelanders (1.29 × 10^-8^) (Jónsson et al., 2017), Danish (1.28 × 10^-8^) (Maretty et al., 2017), a large cattle population (1.2 × 10^-8^) (Harland et al., 2017), two goat trios (unpublished data), as well as three family trios of gene-edited sheep^16^ (**Figure 5**). These findings further supported the conclusion that the *de novo* SNVs in the F1 animals are generated naturally rather than induced by genetic modification.

The CRISPR based approach relies on micro-injection of recombinant Cas9 mRNA/protein and guide RNAs and often results in off-target mutagenesis (Fu et al., 2013; Cho et al., 2014). Off-target sites predicted by different computational programs may have less overlaps (Tsai et al., 2015). In the present study, Cas-OT recognized most of the off-targets that predicted by Cas-OFFinder, indicating the overlapped off-target sites are most likely represent the bona fide off-target sites for further analysis. Furthermore, our work presents trio-based WGS analysis to examine genome-wide *de novo* variants that may be induced by genetic modification in the F1 animals. Consistent with the off-target mutations observed in human cells (Veres et al., 2014; Yang et al., 2014), and mice (Iyer et al., 2015), we observed low incident nuclease induced mutations in large animal models through deep sequencing. Therefore, supported the reliability of CRISPR approach for the production of viable animals.

We have previously demonstrated that disruption of *MSTN* in goats resulted in increased body weight and enlarged myofiber diameters in muscles (Wang et al., 2018b), we also show disruption of the *FGF5* genes led to longer hair fibers in goats (Wang et al., 2016). To test the effect of Cas9-modification on global transcriptional status in the F1 progenies, we conducted RNA-seq on muscle and skin tissues to independently characterize the transcriptional consequences and genetic mechanism by knockout *MSTN* and *FGF5* in F1 progenies. Inconsistent with previous studies (Wang et al., 2016, 2018b), we did not observe significantly expression changes of both *MSTN* and *FGF5* with RNA-seq and validated by qPCR (**Figures 3E,F**). The plausible reason for this difference is animals from two generations were used for analyses. However, in the present study, we did identify a list of DEGs that are known to be associated with muscle development (e.g., *FMOD*, *ARG2*, *TNMD*, *CSRP3*, *PCK2*, *EGR1*, and *TNC*) in the muscle tissues of *MSTN*-disrupted animals, or skin and hair follicle development (*AQP3*, *AQP5*, *SPINK7*, and *WIF1*) in the skin tissues of *FGF5*-disrupted animals. Interestingly, functional enrichment analyses indicated that the DEGs are over-represented in GO terms associated with muscle fiber development in *MSTN*-disrupted goats, and GO terms related to skin development and immune responses in *FGF5*-edited animals. *MSTN* is primarily thought to inhibit muscle differentiation and growth (Poncelet, 1997; Mosher et al., 2007), while *FGF5* represses hair growth by blocking dermal papilla cell activation (Hebert et al., 1994; Ota et al., 2002). Therefore, it is plausible that disruption of these key regulators triggers multiple functional regulatory genes at post-transcriptional levels and eventually resulting in observed phenotypic changes.

## Conclusion

In summary, the present study provides a comprehensive analysis of genomes from edited animals and their progenies through deep sequencing. We provide sufficient evidence to show that the incidence of *de novo* mutations is low not only in edited founder animals (Wang et al., 2018a), but also in the F1 progenies, and their mutation rate is not different from what normally occurs in wild type animals as spontaneous mutations. This study will serve as a valuable resource for evaluating the reliability of the CRISPR-based genome editing technologies in the engineering the genomes of large animals.

## Data Availability

All relevant results are within the paper and its **Supplementary data files**. The raw WGS data of 11 animals are available at the NCBI-SRA under accession nos. SRR6378093, SRR6378094, SRR6378095, SRR6378096, SRR6378097, SRR6378098, SRR6378099, SRR6378100, SRR6378101, SRR6378102, and SRR6378103. Transcriptomic data are available at NCBI-SRA under accession nos. SRR6411189, SRR6411190, SRR6411191, SRR6411192, SRR6411193, SRR6411194, SRR6411195, SRR6411196, SRR6411197, SRR6411198, SRR6411199, SRR6411200, and SRX743626.

## Author Contributions

XW, TS, BP, XH, YJ, and YC conceived the research plans. CL, SZ, YL, GL, YD, LL, and JL performed the experiments. LQ provided the samples. XW, TS, and BP wrote the article.

## Conflict of Interest Statement

The authors declare that the research was conducted in the absence of any commercial or financial relationships that could be construed as a potential conflict of interest. The handling Editor declared a shared affiliation, though no other collaboration, with the authors XW, CL, SZ, YL, GL, YD, LL, JL, YJ, and YC at time of review.

## Footnotes

1 http://www.novogene.com
