# Supplementary material for: Trio-Based Deep Sequencing Reveals a Low Incidence of Off-Target Mutations in the Offspring of Genetically Edited Goats
Source: Front Genet. 2018 Oct 4;9:449. doi: 10.3389/fgene.2018.00449 (PMC6190895; doi:10.3389/fgene.2018.00449)
Supplement: Supplementary file 1 [file Data_Sheet_1.docx]

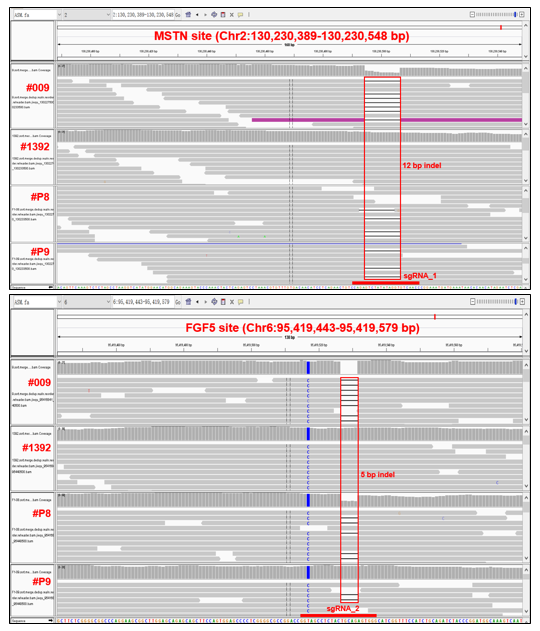
**Figure S1**. The IGV view of on target sites from the *MSTN* _sg1 site and the *FGF5*_sg2 site. Images were generated by the Integrative Genomics Viewer (IGV) browser (http://software.broadinstitute.org/software/igv/).

**
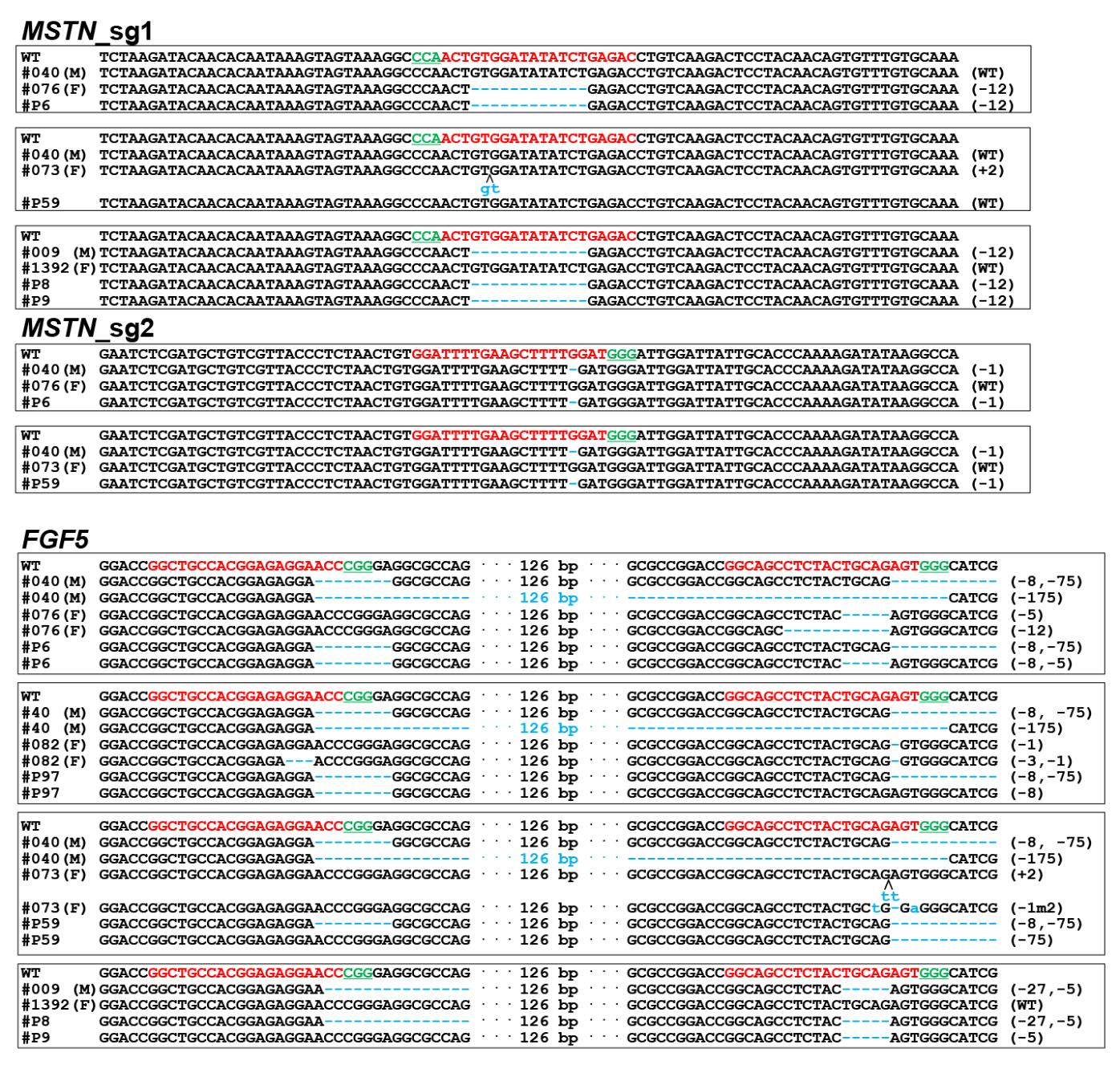
**

**Figure S2.** Validation of the genotypes at target sites in each family trio. Target sequences complementary to sgRNAs of targeted genes are in red text, while the PAM sequences are marked in green. The mutations are marked in blue, dashlines indicate deletions, and lowercases indicate insertions or replacements. Deletions (−) and mutations (m) are shown to the right of each allele.


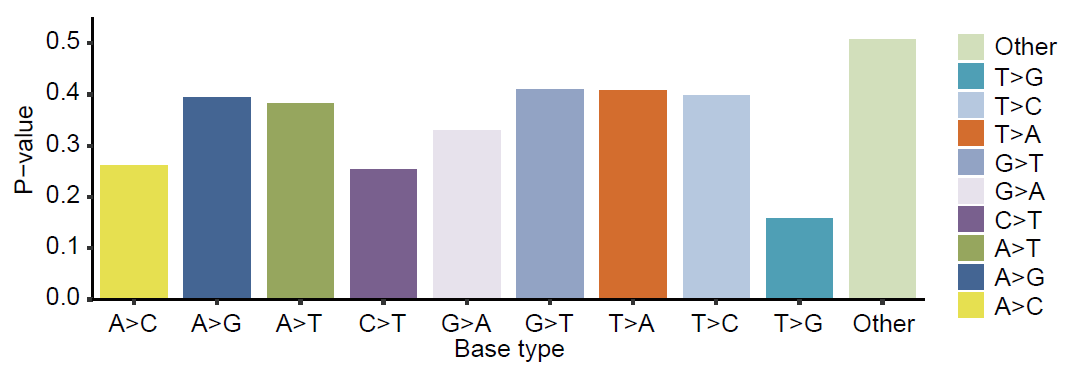
**Figure S3.** The proportion of base changes for each base type in parents and offspring.

**
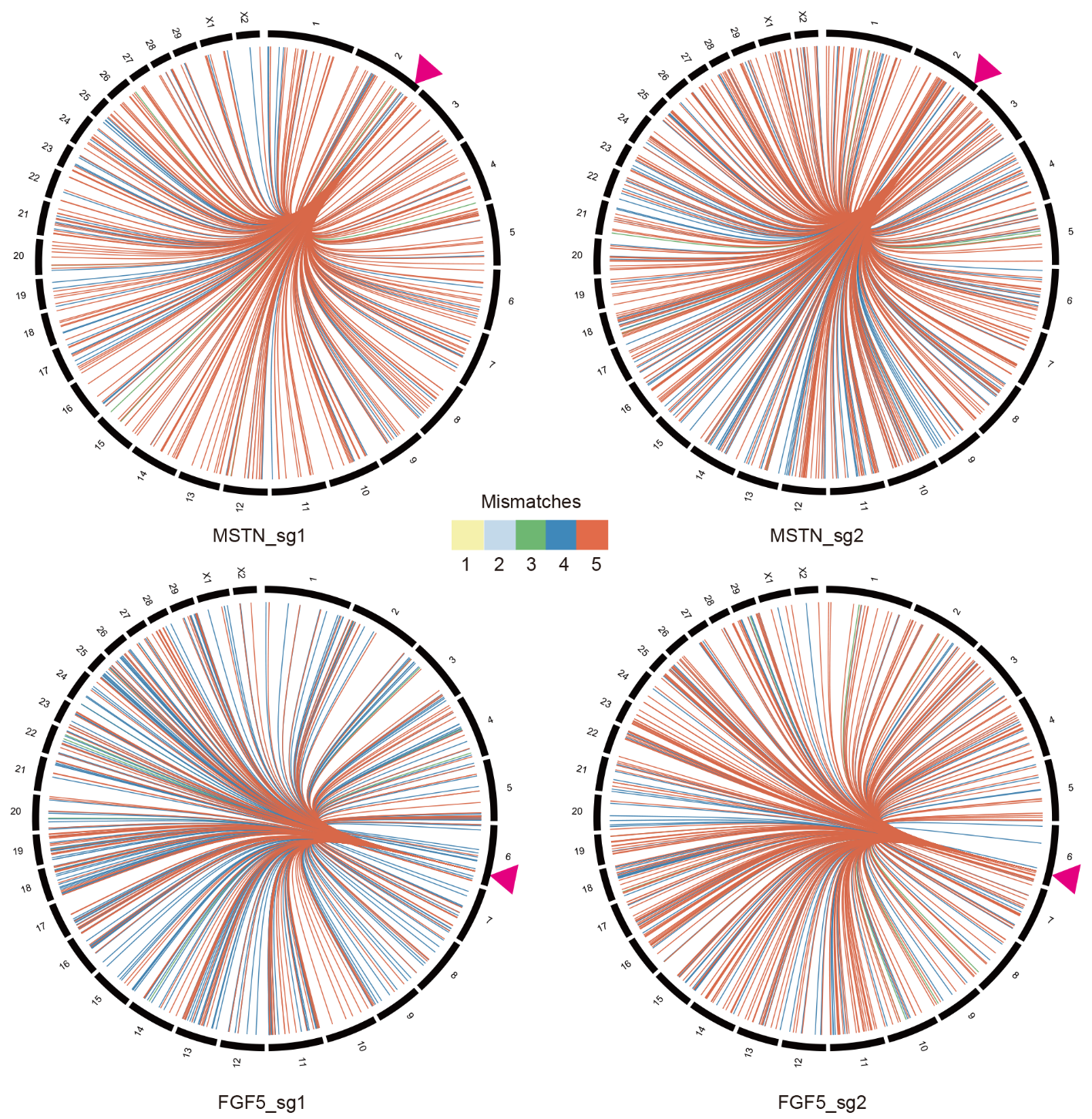
**

**Figure S4.** Distribution of predicted off-target sites for each target at the genome scale. The off-target sites were predicted with the CasOT and Cas-OFFinder. The mismatches of off-target sites, demonstrating with different colors, were allowed to be 5 mis-matches. Pink triangles indicate the position of targeted sites.


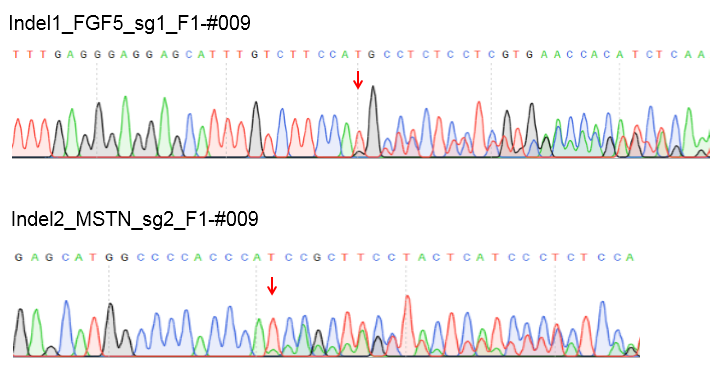


**Figure S5.** Validation of two off-target mutations with Sanger sequencing.


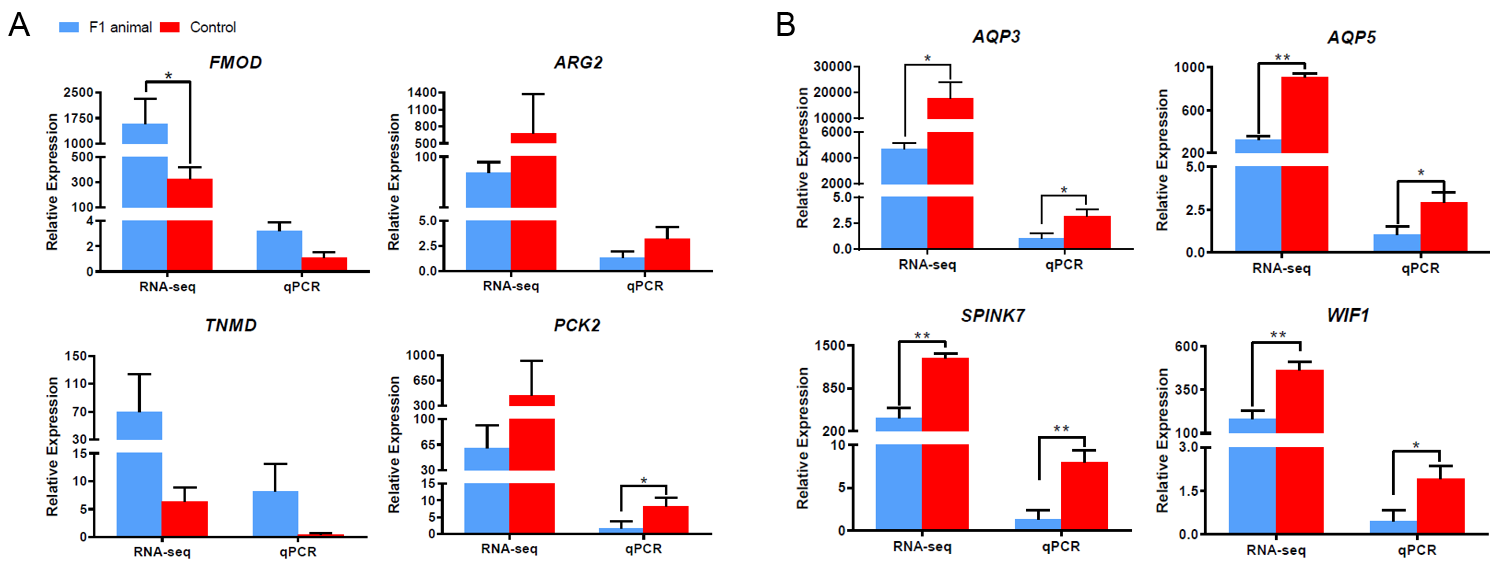


**Figure S6.** Validation of selected DEGs identified by RNA-seq. (A) Validation of *FMOD*, *ARG2*, *TNMD*, and *PCK2* in goat muscles. (B) Validation of *AQP3*, *AQP5*, *SPINK7*, and *WIF1* in goat skins.
